# Feeling of Safety and Comfort towards a Socially Assistive Unmanned Aerial Vehicle That Monitors People in a Virtual Home

**DOI:** 10.3390/s21030908

**Published:** 2021-01-29

**Authors:** Lidia M. Belmonte, Arturo S. García, Rafael Morales, Jose Luis de la Vara, Francisco López de la Rosa, Antonio Fernández-Caballero

**Affiliations:** 1 Departamento de Ingeniería Eléctrica, Electrónica, Automática y Comunicaciones, Universidad de Castilla-La Mancha, 02071 Albacete, Spain; LidiaMaria.Belmonte@uclm.es (L.M.B.); rafael.morales@uclm.es (R.M.); 2 Instituto de Investigación en Informática de Albacete, Universidad de Castilla-La Mancha, 02071 Albacete, Spain; arturosimon.garcia@uclm.es (A.S.G.); JoseLuis.delaVara@uclm.es (J.L.d.l.V.); Fco.Lopez19@alu.uclm.es (F.L.d.l.R.); 3 Departamento de Sistemas Informáticos, Universidad de Castilla-La Mancha, 02071 Albacete, Spain; 4 Biomedical Research Networking Center in Mental Health (CIBERSAM), 28016 Madrid, Spain

**Keywords:** unmanned aerial vehicle (uav), social robot, feeling of safety and comfort, trajectory planning, virtual reality, MATLAB/Simulink, MQTT

## Abstract

Unmanned aerial vehicles (UAVs) represent a new model of social robots for home care of dependent persons. In this regard, this article introduces a study on people’s feeling of safety and comfort while watching the monitoring trajectory of a quadrotor dedicated to determining their condition. Three main parameters are evaluated: the relative monitoring altitude, the monitoring velocity and the shape of the monitoring path around the person (ellipsoidal or circular). For this purpose, a new trajectory generator based on a state machine, which is successfully implemented and simulated in MATLAB/Simulink^®^, is described. The study is carried out with 37 participants using a virtual reality (VR) platform based on two modules, UAV simulator and VR Visualiser, both communicating through the MQTT protocol. The participants’ preferences have been a high relative monitoring altitude, a high monitoring velocity and a circular path. These choices are a starting point for the design of trustworthy socially assistive UAVs flying in real homes.

## 1. Introduction

Over the last decade, there has been an ever growing interest in human–robot interaction (HRI) not only in traditional industrial fields but also in emerging areas such as homes [1]. Of note, personal aerial robotics is becoming more and more pervasive to our home environments. Therefore, it seems vital to understand how home drones are perceived and understood by inhabitants to be fully accepted [2]. Within the broad class of personal robots, assistive robots for the elderly use to be grouped into rehabilitation and socially assistive robots [3]. The manner in which people accept social assistive robots in their life is still unknown [1]. Moreover, one-third of assistive technologies are abandoned within one year of use [4]. For this reason, the acceptability of social assistive robots is an essential aspect to overcome the resistance toward them. This is why acceptance tests for assistive robots caring of elder adults are highly demanded [3,4].

Social robot capabilities include approaching people in a natural manner [5] employing affective elements close to human–human interactions [6]. In this sense, the concept of trust is very important in the adoption of technologies to assist older adults at home [7,8]. Trust can be defined as an attitudinal judgement of the degree to which a user (the ageing adult) can rely on an agent (the social assistive robot) to achieve its goals under conditions of uncertainty [9]. People are more reluctant to engage with robots if negative consequences are more likely, and once confidence has been lost, people take longer to use this technology again [6,10]. Moreover, the safety and efficiency of HRI collaboration often depend on appropriately calibrating trust towards the robot [9] and using a user-centred approach to realise what impacts the development of trust [11]. To date, trust regarding older adults’ adoption of assistive technology has been determined in several ways, including whether the elderly feels safe and comfortable with the proposed solution [12,13,14,15]. The evaluation of these variables requires advanced physical prototyping or, as an alternative, virtual reality (VR) as a simulation tool that allows for fast, flexible and iterative testing processes [16,17,18].

This paper deals with the use of unmanned aerial vehicles (UAVs) as socially assistive robots for dependent people, including ageing adults [19,20]. The design and implementation of the UAV is being done step by step. At each stage of progress, the focus is on ascertaining the acceptance of the final beneficiary regarding the use of the UAV. Considering the characterisation of socially interactive robots [21], the UAV, at this stage of design, includes the perception of emotions, the consideration of natural cues and social competencies such as adaptation to the needs of the users. Other features will be implemented in the future, e.g., communication through high-level dialogue and display of personality and distinctiveness. The purpose of our system is therapeutic help, and therefore assistance.

As it is essential to build a relationship of trust between the person and the flying assistive robot towards a full acceptance from the perspective of the assisted human, it is fundamental to consider the comfort and well-being of the end user. In this sense, the social UAV must carry out its mission, helping the person, but interrupting their daily routine as little as possible. The UAV should be seen as positive and not as a hindrance or even a danger to the person. More concretely, this paper introduces an assistive UAV for home care of dependent people. The mission of the robot is to perform a monitoring flight from time to time to determine their condition [22] and the possible assistance. This monitoring flight basically consists of a series of manoeuvres to take-off, get close to the person, fly around them to obtain facial images and then return to its base. This way, the main interaction between the socially assistive UAV and the person resides in the central part of the monitoring process, the flight around the assisted person. A flying assistance robot could be an effective surveillance solution for a dependent person, since it can be programmed to search and capture data without depending on the person to activate any emergency device. Since it can move to where the person is, it is possible that it will be more successful in its vigilance than multiple cameras installed in fixed places in the house. Finally, the social aspect of the robot can contribute to user acceptance.

The present study is solely focused on analysing three key parameters of the monitoring process: (i) the relative flight height, (ii) the speed of the UAV during the lap to the person, and finally, (iii) the shape of the trajectory that the UAV follows them around, considering two main options, namely, a circular path, which leads to maintain a constant distance between the person and the UAV, and an ellipsoidal one where the distance changes along the way. It should be noted that it was necessary to develop a new algorithm of trajectory planning for a quadrotor model, whose mathematical basis is explained in detail in this paper, to implement these options. This way, the article presents the results of a VR-based trust study considering feelings of safety and comfort on the trajectories followed by a socially assistive quadrotor while monitoring a person.

In order to evaluate the users’ sense of safety and comfort, a survey was conducted in which the participants assessed the three parameters of the UAV’s trajectory during the monitoring process in a VR environment. The conclusions of this study will allow us to continue our challenging research project on assistive UAVs to advance in home care of dependent people. The used VR platform consists of two modules interconnected by means of the message queuing telemetry transport (MQTT) protocol. The modules are (i) a complete UAV simulator that includes the trajectory generator for the monitoring process, the control scheme and the dynamic model of a quadrotor, and, (ii) a VR visualiser in which a quadrotor’s 3D model performs the monitoring process of an avatar in a virtual home. This platform has allowed experiencing in first-person the UAV’s monitoring process, i.e., observing different monitoring paths for the sake of commenting which one feels more comfortable as well as evaluating other aspects regarding safety and privacy. In the simulated scene, the person is completely still, looking forward, being aware of the flight of the UAV around them. In future simulation versions, the level of realism in terms of daily life scenes will be increased. The person will no longer be standing still in a certain (and known) position, but will be allowed to move freely around their home. Neither does this version of the simulator consider obstacles (fixed or mobile) that the UAV may encounter during flight in the real world. Obviously, this issue will soon be addressed using computer vision techniques [23,24].

The remainder of the article is structured as follows. Section 2 describes the main characteristics of the overall system including the VR platform. Section 3.1 details the mathematical development of the new ellipsoidal trajectory planner implemented to carry out the study with the participants. Section 4 details the safety and comfort evaluation study regarding the monitoring parameters studied. Section 5 offers the results of the study. Finally, Section 6 summarises the conclusions of the work and introduces some future research lines.

## 2. General Description of the System

The proposed socially assistive robot’s main mission is to serve as a tool for in-home monitoring of a dependent person in order to enable recording of images that can be analysed to determine their condition and the possible assistance that would be needed. This way, it is possible to provide the patient with help or support and improve their quality of life. This is a quite new area of investigation. In this respect, we could highlight a project for a custom-made quadcopter for monitoring patients indoors using images, sonar and voice recognition techniques [25]. A recent paper has introduced some of the most recent and interesting applications that drones can find in creating ambient assisted living environments for the elderly [26]. Even, a framework has been proposed for UAV navigation and people/object detection in cluttered indoor environments.

As mentioned above, the monitoring process basically consists in a flight around the person’s position to obtain images of the face. Indeed, several very recent papers are dedicated to the still challenging task of designing robust flight controllers to control small flying vehicles (e.g., [27,28,29]), especially indoors [30]. In our case, during the flight around the person, a visual interaction between the robot and the assisted person is produced. The objective of this work is to analyse how this monitoring path should be in order to elicit the highest degree of safety and comfort feelings in the monitored person, which is an imperative issue for indoor drones [31]. For this reason, three key parameters, which are detailed below, are proposed.

**Figure 1:**
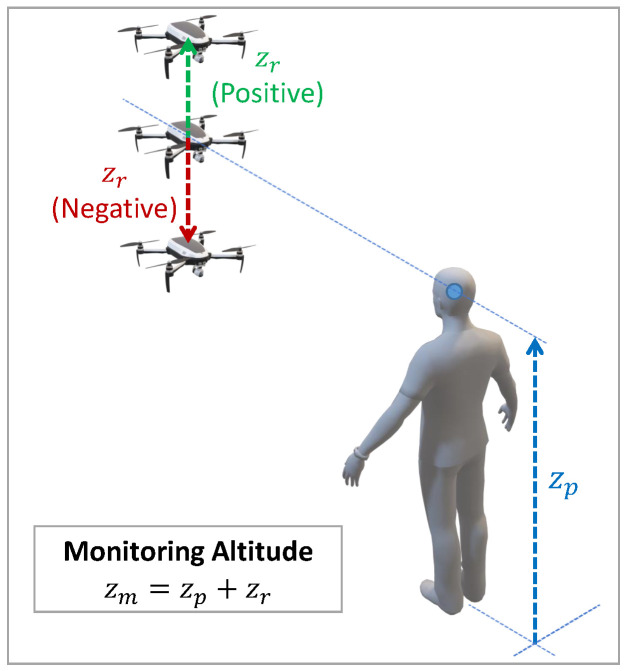
3D representation in isometric perspective of the UAV’s monitoring altitude, $z_{m}$, which is determined by the person’s height, $z_{p}$, plus the relative monitoring altitude, $z_{r}$.

The first parameter is the relative monitoring altitude ($z_{r}$), that is, the height with respect to the monitored person’s head ($z_{p}$) at which the UAV performs the supervisory flight, i.e., the monitoring altitude will be given by expression $z_{m}=z_{p}+z_{r}$. As shown in Figure 1, the relative altitude parameter can be positive, negative, or even null, giving rise to three possible options in the monitoring process: the UAV is located (i) above ($z_{r}>0$), (ii) at ($z_{r}=0$), and, (iii) below ($z_{r}<0$) the person’s head height.

**Figure 2:**
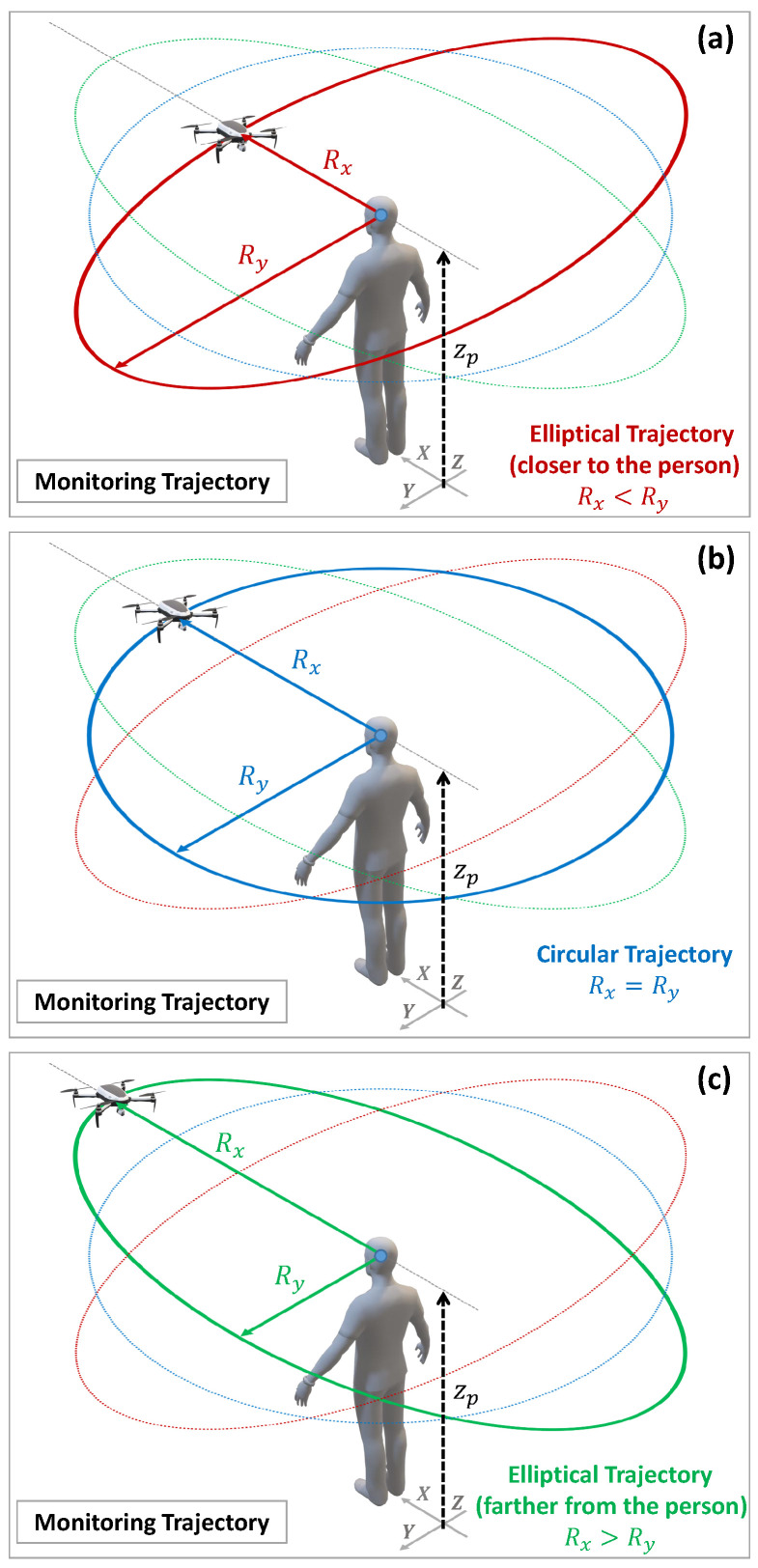
3D representation in isometric perspective of the trajectory according to the UAV’s monitoring radius: (**a**) elliptical trajectory closer to the person’s face ($R_{x}>R_{y}$); (**b**) circular trajectory in which the monitoring radius is constant ($R_{x}=R_{y}$); (**c**) elliptical trajectory farther from the person’s face ($R_{x}<R_{y}$).

The second parameter is the monitoring velocity ($\omega_{m}$), that is, the speed at which the UAV moves during the flight around the assisted person. This velocity will directly influence the monitoring time and is, therefore, a parameter that can greatly influence the feelings of comfort and safety of the monitored person. Finally, the third parameter is the monitoring radius, i.e., the distance at which the UAV is placed from the person’s position during the monitoring flight. This radius can be constant, which leads the UAV to trace a circular path around the person being monitored, or it can be variable, resulting in an elliptical path. For this reason, we also refer to this parameter as the monitoring trajectory (or the shape of the trajectory). Figure 2 shows the three possible options: (a) elliptical trajectory with the UAV closer to the person’s face, (b) circular trajectory with a constant distance between the UAV and the person, and (c) elliptical trajectory but with the UAV away from the person’s face and closer to their side.

As can be inferred from the above parameters, the monitoring process is closely related to the position and orientation of the assisted person. Therefore, the trajectory planner needs to know these two variables at all time. This information would be provided by a module in charge of locating the person and would probably be implemented via computer vision using the camera on board of the UAV. However, even though it is paramount for the functioning of the system, this is out of the scope of the paper and will be addressed in the future. For this paper, we assume that the current position and orientation of the person is available at all times. Based on this information, the planner determines the reference signal for the position and yaw angle of the UAV for the different manoeuvres that make up the entire monitoring process. These references are the entry of the control algorithm that finally calculates the inputs to be applied for the UAV to perform the flight correctly.

**Figure 3:**
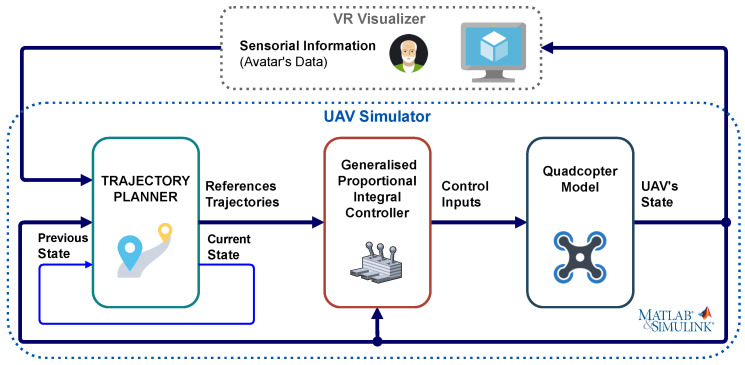
General diagram of the UAV s imulator which receives from the VR visualiser the information concerning the person’s avatar to calculate the reference trajectories used by the controller to guide the UAV in the monitoring process while returns the aircraft’s position and orientation to represent its flight in a virtual home environment.

Figure 3 represents the general scheme of the socially assistive UAV simulator implemented in MATLAB/Simulink^®^. It shows the different blocks that compose it, which are: the dynamic model of a quadrotor UAV, the nonlinear control algorithm, and finally the trajectory planner for the monitoring process. It should be mentioned that: (i) this UAV simulator is part of a VR platform developed by our research group, whose operation is explained in Section 4; (ii) the dynamics of the quadrotor and the generalised proportional integral (GPI) controller, which can be consulted in a previous work [32]. Regarding the trajectory planner, the initial versions [16,33] were designed for a circular monitoring path at a constant height defined by the person’s head. However, to carry out the study of the influence of the above-mentioned parameters in the safety and comfort of the monitored user, it has been necessary to implement a new trajectory planner algorithm which will be detailed next.

## 3. Trajectory Planning

This section describes the new ellipsoidal trajectory planning algorithm for the monitoring flight of a quadrotor UAV. It consists of a state machine in which the reference signals for the UAV’s position and yaw angle are defined in each of the manoeuvres necessary to complete the monitoring process. It should be recalled that the signals generated by the path planner are the (reference) inputs of the UAV’s controller which in the end calculates what (control) inputs should be applied to the aircraft to direct its flight correctly during the monitoring of the person. In this process, the information captured by the camera on board the UAV will be sent to a processing station for analysis using artificial intelligence techniques, such as emotion recognition [34,35,36], to determine the person’s physical condition and possible assistance [37].

Before detailing the equations of the different states that make up the planner, we proceed to define the mathematical basis for plotting the ellipsoidal path around the person (an ellipse on the horizontal plane defined by the monitoring height, centred on the person’s position and rotated according to their orientation).

### 3.1. Equations of the Ellipse

Firstly, the main path of the monitoring process consists in an ellipse in the horizontal plane (XY) whose mathematical equation in the simplest case (centred at the origin) is the following:

(1) $$\frac{x^{2}}{R_{x}^{2}}+\frac{y^{2}}{R_{y}^{2}}=1$$

where $R_{x}$ is the radius on the *X* axis and $R_{y}$ is the radius on the *Y* axis.

However, we must consider that the ellipse will be centred on the position of the person, therefore its equation would be as follows:

(2) $$\frac{{\left(x-C_{x}\right)}^{2}}{R_{x}^{2}}+\frac{{\left(y-C_{y}\right)}^{2}}{R_{y}^{2}}=1$$

where $R_{x}$ and $R_{y}$ are the radius on the *X* and *Y* axes, respectively, and $\left(C_{x},C_{y}\right)$ are the coordinates of the centre of the ellipse that will coincide with the person’s horizontal position, $\left(x_{p},y_{p}\right)$.

Moreover, we must consider that the ellipse will also be rotated an angle of θ rad, according to the orientation of the person ($\alpha_{p}$), and therefore the equation will be expressed as follows:

(3) $$\begin{array}{c} \frac{{\left(\left(x-C_{x}\right)\cdot \cos\left(\theta \right)+\left(y-C_{y}\right)\cdot \sin\left(\theta \right)\right)}^{2}}{R_{x}^{2}}+\frac{{\left(\left(x-C_{x}\right)\cdot \sin\left(\theta \right)-\left(y-C_{y}\right)\cdot \cos\left(\theta \right)\right)}^{2}}{R_{y}^{2}}=1 \end{array}$$

where $R_{x}$ and $R_{y}$ are the radius on the *X* and *Y* axes, respectively, $\left(C_{x},C_{y}\right)$ are the coordinates of the centre of the ellipse, and θ is the above-mentioned rotation angle.

**Figure 4:**
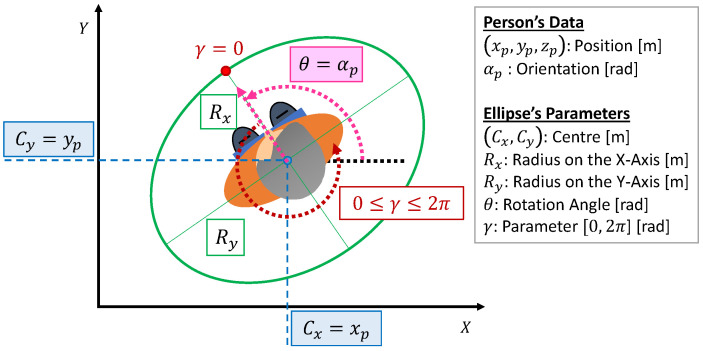
Variables of the parametric equation of the ellipse centred at the XY position of the person and rotated according to their orientation.

To conclude, Equation (4) details the parametric formula depending on the variable γ of an ellipse centred at the position $\left(C_{x},C_{y}\right)$, and rotated at a certain angle θ. Figure 4 represents this parameterisation in the particular case of the monitoring process, i.e., when the ellipse is centred and rotated according to the person’s position and orientation, respectively ($\left(C_{x},C_{y}\right)=\left(x_{p},y_{p}\right);\theta =\alpha_{p}$). This parametric equation will be useful for the generation of the reference path for the UAV’s XY position as will be explained bellow.

(4) $$\begin{array}{ccc} x(\gamma ) & = & C_{x}+R_{x}\cos\left(\gamma \right)\cos\left(\theta \right)-R_{y}\sin\left(\gamma \right)\sin\left(\theta \right)\\ y(\gamma ) & = & C_{y}+R_{x}\cos\left(\gamma \right)\sin\left(\theta \right)+R_{y}\sin\left(\gamma \right)\cos\left(\theta \right) \end{array}$$

where:

- $C_{x}$ is the X-coordinate of the centre [m],
- $C_{y}$ is the Y-coordinate of the centre [m],
- $R_{x}$ is the radius on the X-axis [m],
- $R_{y}$ is the radius on the Y-axis [m],
- θ is the rotation angle [rad], and
- γ is the parameter, which ranges from 0 to 2π.

### 3.2. Determination of the Safety Position

**Figure 5:**
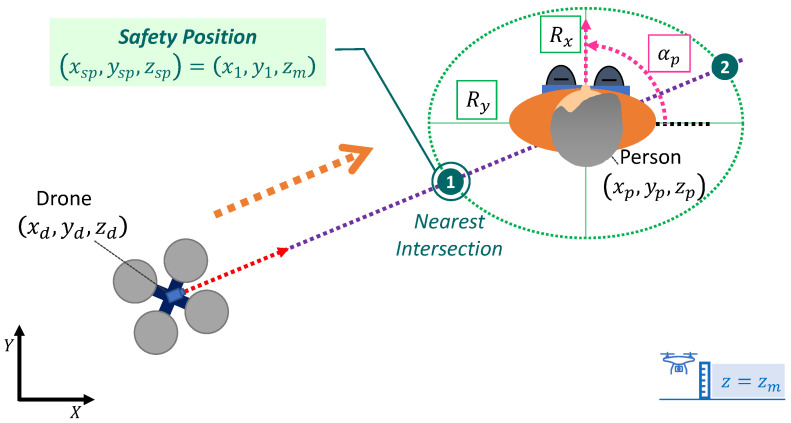
Determination of the safety position $\left(x_{sp},y_{sp},z_{sp}\right)$ to which the UAV is approaching to start the elliptical monitoring lap around the person.

The safety position is the point at which the UAV approaches after take-off (to approximate the patient’s immediate surroundings) and from which it begins to rotate around the person to take images that will be sent for analysis. After completing the data capture lap, the UAV returns from this safety position to the base position for landing. Therefore, the safety position is an important way-point in the monitoring process whose calculation is explained next. In the case of considering an ellipsoidal monitoring trajectory, the XY coordinates of the safety position will be determined by the nearest intersection between the imaginary line connecting the centre of the person and the position of the UAV with the ellipse centred and rotated according to the person’s position and orientation (see Figure 5). It should be recalled that the *Z* coordinate of this safety position will be given by the UAV’s altitude in the monitoring process (person’s head height plus relative altitude parameter).

This way, the problem raised is: (1) determine the points of intersection of a line passing through the centre of the ellipse and the position of the UAV with the ellipse rotated and centred on the person; (2) determine the point of intersection closest to the position of the UAV. The mathematical resolution is detailed below.

#### 3.2.1. Calculation of the Intersection Points with the Rotated Ellipse

The equation of a line defined by two points, $\left(x_{a},y_{a}\right)$ and $\left(x_{b},y_{b}\right)$ is detailed in Equation (5).

(5) $$y=y_{a}+\frac{y_{b}-y_{a}}{x_{b}-x_{a}}\cdot \left(x-x_{a}\right);$$

The above can also be expressed in parametric form, thus giving rise to two individual expressions for each component of the horizontal plane (XY) dependent on the *t* parameter:

(6) $$\begin{array}{ccc} x(t) & = & x_{a}+\left(x_{b}-x_{a}\right)\cdot t;\\ y(t) & = & y_{a}+\left(y_{b}-y_{a}\right)\cdot t; \end{array}$$

If the point $\left(x_{a},y_{a}\right)$ matches with the centre of the ellipse around the person $\left(x_{p},y_{p}\right)$, and the point $\left(x_{b},y_{b}\right)$ is the UAV’s position, $\left(x_{d},y_{d}\right)$, the parametric Equation (6) can be written as follows:

(7) $$\begin{array}{ccc} x(t) & = & x_{p}+\left(x_{d}-x_{p}\right)\cdot t;\\ y(t) & = & y_{p}+\left(y_{d}-y_{p}\right)\cdot t; \end{array}$$

In order to calculate the intersection between the line defined by Equation (6) and the ellipse defined by the person’s pose, i.e., centred and rotated according to their position and orientation, $\left(C_{x},C_{y}\right)=\left(x_{p},y_{p}\right);\theta =\alpha_{p}$, it is necessary to substitute Equation (7) in Equation (3), obtaining:

(8) $$\begin{array}{c} \frac{{\left(\left(x_{p}+\left(x_{d}-x_{p}\right)\cdot t-x_{p}\right)\cdot \cos\left(\alpha_{p}\right)+\left(y_{p}+\left(y_{d}-y_{p}\right)\cdot t-y_{p}\right)\cdot \sin\left(\alpha_{p}\right)\right)}^{2}}{R_{x}^{2}}+\\ \frac{{\left(\left(x_{p}+\left(x_{d}-x_{p}\right)\cdot t-x_{p}\right)\cdot \sin\left(\alpha_{p}\right)-\left(y_{p}+\left(y_{d}-y_{p}\right)\cdot t-y_{p}\right)\cdot \cos\left(\alpha_{p}\right)\right)}^{2}}{R_{y}^{2}}=1 \end{array}$$

(9) $$\begin{array}{c} \frac{{\left(\left(\left(x_{d}-x_{p}\right)\cdot t\right)\cdot \cos\left(\alpha_{p}\right)+\left(\left(y_{d}-y_{p}\right)\cdot t\right)\cdot \sin\left(\alpha_{p}\right)\right)}^{2}}{R_{x}^{2}}+\\ \frac{{\left(\left(\left(x_{d}-x_{p}\right)\cdot t\right)\cdot \sin\left(\alpha_{p}\right)-\left(\left(y_{d}-y_{p}\right)\cdot t\right)\cdot \cos\left(\alpha_{p}\right)\right)}^{2}}{R_{y}^{2}}=1 \end{array}$$

After some operations, Equation (9) is simplified and the result is detailed in Equation (10):

(10) $$\frac{t^{2}\cdot f_{1}}{R_{x}^{2}}+\frac{t^{2}\cdot f_{2}}{R_{y}^{2}}=1$$

where

$$\begin{array}{ccc} f_{1} & = & \left(x_{d}^{2}+x_{p}^{2}-2x_{d}x_{p}\right)\cdot \cos^{2}\left(\alpha_{p}\right)+\left(y_{d}^{2}+y_{p}^{2}-2y_{d}y_{p}\right)\cdot \sin^{2}\left(\alpha_{p}\right)+\\ & & 2\cdot \left(x_{d}-x_{p}\right)\cdot \left(y_{d}-y_{p}\right)\cdot \cos\left(\alpha_{p}\right)\cdot \sin\left(\alpha_{p}\right);\\ f_{2} & = & \left(x_{d}^{2}+x_{p}^{2}-2x_{d}x_{p}\right)\cdot \sin^{2}\left(\alpha_{p}\right)+\left(y_{d}^{2}+y_{p}^{2}-2y_{d}y_{p}\right)\cdot \cos^{2}\left(\alpha_{p}\right)-\\ & & 2\cdot \left(x_{d}-x_{p}\right)\cdot \left(y_{d}-y_{p}\right)\cdot \sin\left(\alpha_{p}\right)\cdot \cos\left(\alpha_{p}\right); \end{array}$$

Now, from Equation (10) it is possible to obtain the expression of the parameter *t* (see Equation (11)), and from that determine two possible solutions, $t_{1}$ and $t_{2}$, using Equations (12) and (13), respectively:

(11) $$t=\frac{R_{x}\cdot R_{y}}{\sqrt{f_{1}\cdot R_{y}^{2}+f_{2}\cdot R_{x}^{2}}}$$

- Solution 1:
  (12) $$t_{1}=\frac{R_{x}\cdot R_{y}}{\sqrt{f_{1}\cdot R_{y}^{2}+f_{2}\cdot R_{x}^{2}}}$$
- Solution 2:
  (13) $$t_{2}=-t_{1}=-\frac{R_{x}\cdot R_{y}}{\sqrt{f_{1}\cdot R_{y}^{2}+f_{2}\cdot R_{x}^{2}}}$$

Substituting the two solutions of the *t* parameter, $t_{1}$ and $t_{2}$, in the parametric Equation (7), we obtain the two points of intersection between the rotated ellipse and the line joining the position of the UAV with the centre of the ellipse (the position of the person):

- Solution 1:
  (14) $$\begin{array}{ccc} x_{1}=x\left(t_{1}\right) & = & x_{p}+\left(x_{d}-x_{p}\right)\cdot t_{1};\\ y_{1}=y\left(t_{1}\right) & = & y_{p}+\left(y_{d}-y_{p}\right)\cdot t_{1}; \end{array}$$
- Solution 2:
  (15) $$\begin{array}{ccc} x_{2}=x\left(t_{2}\right) & = & x_{p}+\left(x_{d}-x_{p}\right)\cdot t_{2};\\ y_{2}=y\left(t_{2}\right) & = & y_{p}+\left(y_{d}-y_{p}\right)\cdot t_{2}; \end{array}$$

#### 3.2.2. Determination of the Nearest Intersection Point (Safety Position)

Finally, to determine the safety position $\left(x_{sp},y_{sp}\right)$, it is necessary to know which intersection point is the closest to the position of the UAV (see example in Figure 5). To do this, we calculate the distance between the UAV and the two candidates calculated above:

(16) $$\begin{array}{ccc} d_{1} & = & \sqrt{{\left(x_{1}-x_{d}\right)}^{2}+{\left(y_{1}-y_{d}\right)}^{2}};\\ d_{2} & = & \sqrt{{\left(x_{2}-x_{d}\right)}^{2}+{\left(y_{2}-y_{d}\right)}^{2}}; \end{array}$$

Thus, if the distance $d_{1}$ is equal to or less than $d_{2}$, the first point will be the safety position in the monitoring process, $\left(x_{sp},y_{sp}\right)=\left(x_{1},y_{1}\right)$. Conversely, if the distance $d_{2}$ is less than $d_{1}$, the safety position will be the second point, $\left(x_{sp},y_{sp}\right)=\left(x_{2},y_{2}\right)$. Please remember that the *Z* coordinate of this safety position will be given by the altitude of the UAV in the monitoring process, $z_{m}$ (person’s height plus relative height parameter).

### 3.3. Ellipsoidal Trajectory around the Person

To generate the reference ellipsoidal path around the person, it is necessary to convert the safety position at which the UAV stops for a time before turning around him or her into the corresponding value of the parameter γ of the parametric equation of the ellipse, Equation (4). To do this, we first calculate the position in the original ellipse $\left(x_{e_{o}},y_{e_{o}}\right)$, an ellipse without rotation and centred at the point (0,0), to which the safety position corresponds in the rotated and person-centred ellipse, $PosE_{Rot}=\left(x_{sp},y_{sp}\right)$. In this way, and using matrix notation, we obtain:

(17) $$\underset{PosE_{Rot}}{\underset{︸}{\left[\begin{array}{c} x_{sp}\\ y_{sp} \end{array}\right]}}=\underset{MAT_{ROT}}{\underset{︸}{\left[\begin{array}{cc} cos(\alpha_{p}) & -sin(\alpha_{p})\\ sin(\alpha_{p}) & \cos(\alpha_{p}) \end{array}\right]}}\underset{PosE_{Orig}}{\underset{︸}{\cdot \left[\begin{array}{c} x_{e_{o}}\\ y_{e_{o}} \end{array}\right]}}+\underset{Centre}{\underset{︸}{\left[\begin{array}{c} x_{p}\\ y_{p} \end{array}\right]}}$$

(18) $$\left[\begin{array}{c} x_{e_{o}}\\ y_{e_{o}} \end{array}\right]={\left[\begin{array}{cc} cos(\alpha_{p}) & -sin(\alpha_{p})\\ sin(\alpha_{p}) & \cos(\alpha_{p}) \end{array}\right]}^{-1}\cdot \left[\begin{array}{c} x_{sp}-x_{p}\\ y_{sp}-y_{p} \end{array}\right]$$

Secondly, and depending on the quadrant in which the calculated position is located in the original ellipse, $\left(x_{e_{o}},y_{e_{o}}\right)$, the value of the parameter γ is determined using the inverse of the cosine or sine functions as follows:

- Quadrant I: $x_{e_{o}}>0$, $y_{e_{o}}>0$
  (19) $$\gamma =\arccos\left(\frac{x_{e_{o}}}{R_{x}}\right)=\arcsin\left(\frac{y_{e_{o}}}{R_{y}}\right)$$
- Quadrant II: $x_{e_{o}}<0$, $y_{e_{o}}>0$
  (20) $$\gamma =\arccos\left(\frac{x_{e_{o}}}{R_{x}}\right)=\pi -\arcsin\left(\frac{y_{e_{o}}}{R_{y}}\right)$$
- Quadrant III: $x_{e_{o}}<0$, $y_{e_{o}}<0$
  (21) $$\gamma =2\pi -\arccos\left(\frac{x_{e_{o}}}{R_{x}}\right)=\pi -\arcsin\left(\frac{y_{e_{o}}}{R_{y}}\right)$$
- Quadrant IV: $x_{e_{o}}>0$, $y_{e_{o}}<0$
  (22) $$\gamma =2\pi -\arccos\left(\frac{x_{e_{o}}}{R_{x}}\right)=2\pi +\arcsin\left(\frac{y_{e_{o}}}{R_{y}}\right)$$

Once we know the value of the γ parameter to which the initial position correspond and from which the ellipsoidal path around the person will start, that is, the equivalent to the safety position ($\gamma_{initial}=\gamma_{sp}$), we can use the parametric formula of the ellipse, Equation (4), and gradually increase its parameter to complete one lap ($\gamma_{initial}+2\pi$ rad). This will be the procedure used to obtain the reference trajectories for the UAV’s XY position for the ellipse around the person as detailed below.

### 3.4. States of the Trajectory Planner

This section describes in the detail the states that compose the new trajectory planner for the monitoring process considering an ellipsoidal path around the monitored person. In the previous version of the planner [16], the path around the person was circular. It is worth mentioning that this particular case can also be implemented using the equations described below simply by taking $R_{x}=R_{y}$. Compared to the previous version, in which the monitoring flight was only performed at a constant height defined by the person’s head, it is now also possible to vary this height by the above-mentioned parameter of relative monitoring altitude ($z_{r}$). In this way, the UAV will fly at the same height as the person’s head ($z_{r}=0$), at a higher height ($z_{r}>0$), or at a lower height ($z_{r}<0$).

**Figure 6:**
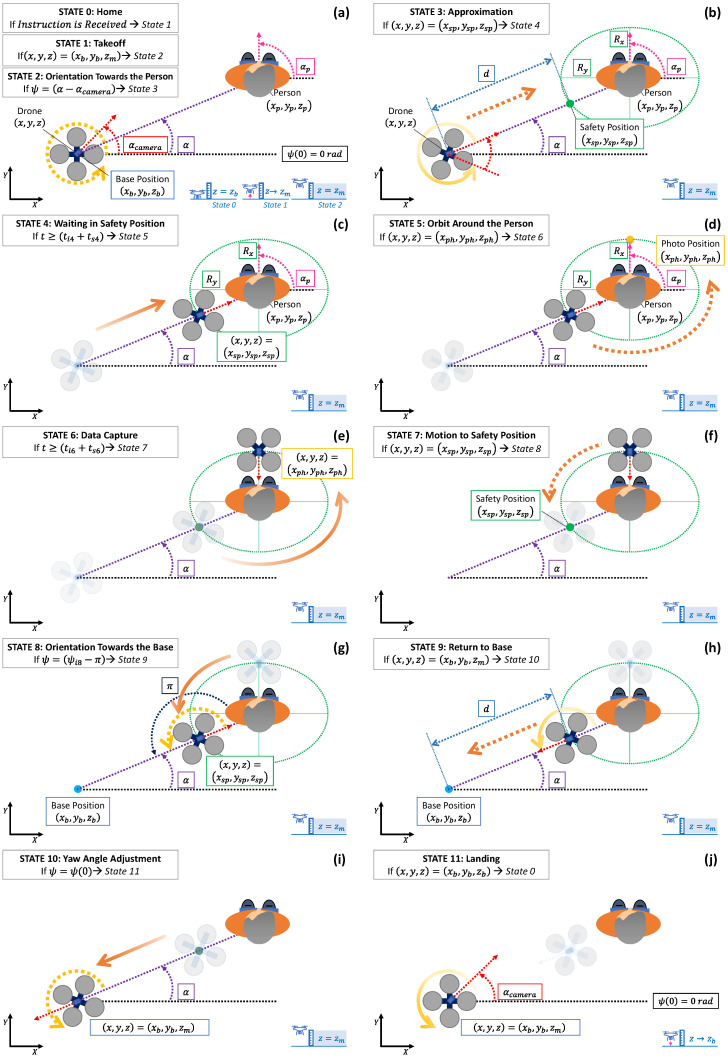
Graphical representation of the trajectory planner’s states: (**a**) states 0—home, 1—takeoff, and 2—orientation towards the person; (**b**) state 3—approximation; (**c**) state 4—waiting in safety position; (**d**) state 5—orbit around the person; (**e**) state 6—data capture; (**f**) state 7—motion to safety position; (**g**) state 8—orientation towards the base; (**h**) state 9—return to base; (**i**) state 10—yaw angle adjustment; (**j**) state 11—landing.

Before describing the states of the planner, the following assumptions have been considered in its development: (i) the UAV remains in its base position before and after a monitoring process $\left(x_{b},y_{b},z_{b}\right)$; (ii) the information of the monitored person’s position, $\left(x_{p},y_{p},z_{p}\right)$, and orientation, $\alpha_{p}$, are known at each moment; (iii) the reference trajectories are defined so that the UAV’s camera always points in its forward direction or towards the person; (iv) there are no energy limitations since the monitoring process is carried out in a short period of time; and, (v) consequently, it is presumed that the person does not walk during this brief process. The states of the trajectory planner are a total of twelve represented graphically in Figure 6 and whose equations for the position and orientation of the UAV are summarised in Table 1 and Table 2. The details of each state are as follows.

- STATE 0—Home: Represents the UAV waiting in its base position, $\left(x_{b},y_{b},z_{b}\right)$, and initially oriented with a yaw angle, ψ(0), which by default is equal to 0 rad. When it receives the instruction to start the monitoring process, it transits to state 1.
- STATE 1—Takeoff: This is the first manoeuvre to raise the UAV, with a constant speed $v_{z}$, from the base level, $z_{b}$, until the monitoring altitude, $z_{m}$. This level will be equal to the person’s height (measured in the centre of their head, $z_{p}$) plus the relative altitude parameter ($z_{r}$), which can be positive, zero, or even negative to consider the three scenarios mentioned before (above their head, coinciding with it or below it). Thus, when the UAV reaches the monitoring level, $z_{m}=z_{p}+z_{r}$, the planner switches to state 2.
- STATE 2—Orientation Towards the Person: After takeoff, the UAV is requested to turn, varying the yaw angle with a speed defined by $\omega_{\psi }$, in order to find the person with its on board camera. Since the person’s position is known at each moment, it is possible to calculate the final (target) yaw angle, $\psi_{f2}$, which will be the difference between the angle $\alpha =arctan(\frac{y_{p}-y_{i2}}{x_{p}-x_{i2}})$ and the camera’s angle $\alpha_{camera}$ as can be observed in Figure 6a. Therefore, the yaw angle is gradually modified until the UAV’s camera is focused on the person. At this moment, the planner transits to state 3.
- STATE 3—Approximation: The UAV must approach the person (already centred on the camera’s view) to turn around them in the next states. As already detailed, the UAV must reach the safety position whose XY coordinates will be given by the nearest intersection of the (imaginary) line joining the positions of the UAV and the person with the ellipse centred and rotated according to their position and orientation, respectively. The relative monitoring altitude should be maintained constant, i.e., the *Z* coordinate of the safety position will match the monitoring level. The planner will transit to state 4 once the UAV reaches the safety position $\left(x_{sp},y_{sp},z_{sp}\right)$.
- STATE 4—Waiting in Safety Position: Intermediate state in which the UAV waits for a short time to start later the lap around the person more precisely. Therefore, once the programmed time ($t_{s4}$) has elapsed, it transits to state 5.
- STATE 5—Orbit Around the Person: At this moment, the ellipsoidal rotation of the UAV around the person starts from the safety position (whose equivalence to the parameter γ of the ellipse’s parametric formula is determined according to Equations (17)–(22)), while the flight height is kept constant. On the other hand, the UAV’s yaw angle is modified during the ellipsoidal trajectory so that its on board camera is always pointing towards the person. This way, once the UAV’s camera finds the person’s face (when the UAV is in front of the face), the planner switches to state 6. Since the person’s information is known, and the ellipse is rotated according to their orientation, $\theta =\alpha_{p}$, the position in front of their face, labelled as photo position in Figure 6d, $\left(x_{ph},y_{ph},z_{ph}\right)$, can be calculated from the ellipse’s parametrisation by taking γ=0 [rad].
- STATE 6—Data Capture: In the general case, the UAV can remain in the position in front of the person’s face, $\left(x_{ph},y_{ph},z_{ph}\right)$, to capture images with better accuracy. Once the data capture timer $\left(t_{s6}\right)$ elapses, the planner transitions to state 7. On the contrary, if the UAV is programmed to take pictures of the person for the entire lap around him or her, the waiting time can be set to zero ($t_{s6}=0$) and the planner would transit directly to state 7. The UAV would continue the lap without stopping.
- STATE 7—Motion and Safety Position: The UAV will continue its ellipsoidal rotation around the monitored person to reach the safety position again. The height of the UAV will be kept constant and the yaw angle will be varied so that the on board camera continues to be pointed at the patient. Mathematically, it is, therefore, equal to state 5 and, consequently, the reference trajectories are determined in a very similar way. Once the UAV reaches the safety position, $\left(x_{sp},y_{sp},z_{sp}\right)$, the planner transits to state 8.
- STATE 8—Orientation Towards the Base: Once the UAV has completed its ellipsoidal trajectory around the monitored person, it is commanded to turn on itself, gradually adjusting its yaw angle to search for the base with its camera. In this way, a procedure similar to state 2 will be followed, but the final yaw angle is now known in advance. As can be seen in Figure 6g, the UAV must turn −π rad, so that the UAV’s camera directly points towards the base. At this point, the planner will switch to state 9.
- STATE 9—Return to Base: The aircraft is ordered to return to over the base position while keeping the flight height constant. In this way, the UAV’s movement is the same as state 3, but in the opposite direction, thus the reference trajectories are calculated in a similar way (in this case even the final position is known, so no additional calculation is necessary unlike state 3 in which the safety position was determined). Once the UAV is positioned over its base, at $\left(x_{b},y_{b},z_{m}\right)$, the planner transits to state 10.
- STATE 10—Yaw Angle Adjustment: Before landing, the yaw angle of the UAV is adjusted to its initial value to prepare the aircraft for future monitoring processes, while the UAV’s position is kept constant. The reference trajectories will be, therefore, analogous to state 2. This way, when the adjustment is completed, the planner will shift to state 11.
- STATE 11—Landing: Finally the UAV is commanded to land at its base ($x_{b},y_{b},z_{b})$. In this way, the aircraft will descend vertically, with a constant speed defined by the parameter $v_{z}$, until reaching the base level, and the planner will transit to the initial state (0) to be ready for the next monitoring process.

### 3.5. Simulation Results

**Figure 7:**
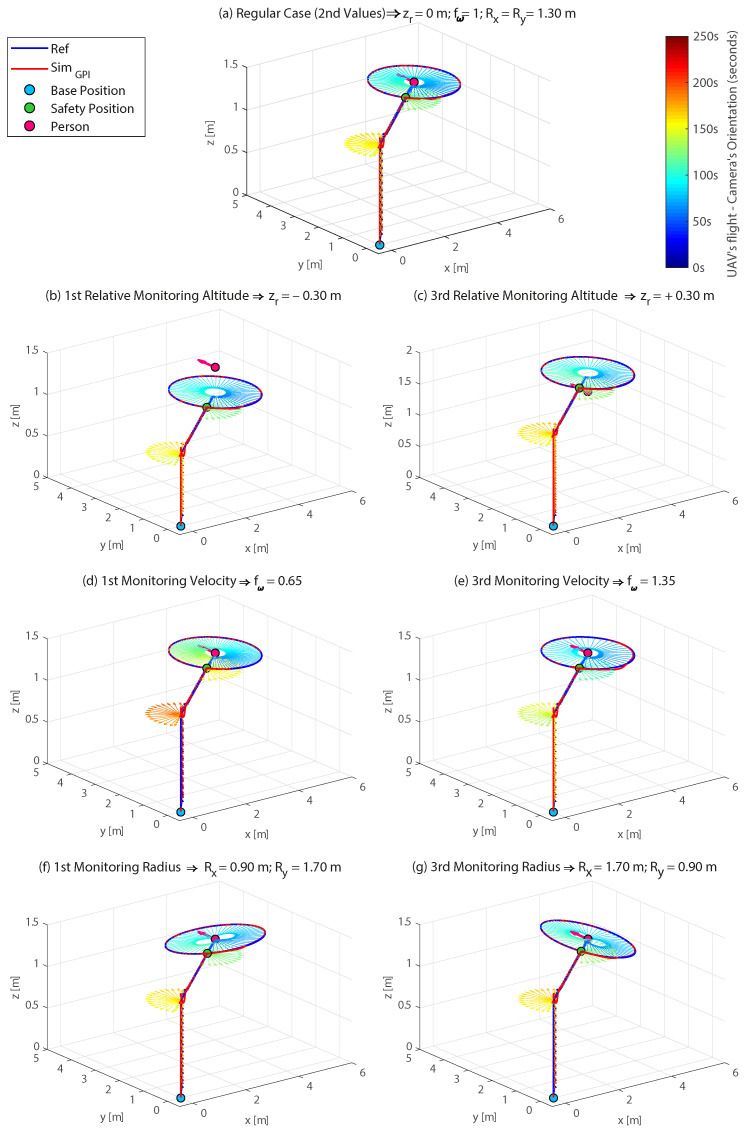
Results of the tests to verify the new ellipsoidal trajectory planner carried out using the UAV simulator (part of the VR platform and implemented in MATLAB/Simulink^®^). For each test, the following is represented: (1) trajectory generated by the planner (in blue) against the actual trajectory performed by the quadrotor model as the result of the action of the GPI controller (in red); (2) orientation of the UAV’s camera by means of arrows whose colour change over time; (3) way-points: base position (blue circle), safety position (green circle), and person’s position and orientation (pink circle and arrow).

The results of the simulation tests carried out using the settings shown in Table 3 and Table 4 are summarised in Figure 7.

Each subfigure (a–g) corresponding to each test performed, represents in 3D the reference path generated by the planner (in blue) and the actual trajectory performed by the UAV (in red) during the monitoring process of a person whose head position and orientation (in pink) are known. In addition, the orientation of the UAV’s camera is also represented by means of arrows whose colours change over time (the colour bar on the right side represents the time evolution during the complete simulation of 250 s). The results show how the trajectory planner is able to correctly generate the reference for the position and yaw angle of the UAV in each case. The accuracy of the GPI controller [32], which calculates the (control) inputs in order to reduce the tracking error of the trajectories to zero, is also checked. This ensures that the quadrotor model performs the monitoring flight accurately and according to the planner’s references.

## 4. Experimental Setup

**Figure 8:**
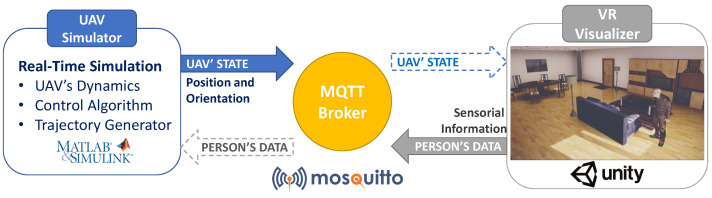
Architecture of the distributed platform.

The system described in this paper relies on the software and hardware platform described in [16]. It is a distributed architecture with two main modules: the UAV simulator, in charge of reproducing the flight of the UAV considering its dynamics, and generating the trajectories; and the VR Visualiser, in charge of rendering the virtual UAV and its behaviour, as well as the virtual environment in which the UAV flight takes place. These two modules communicate with each other using the MQTT protocol, exchanging the position and orientation of the user as well as of the UAV. The user’s information, sent from the VR Visualiser to the UAV simulator, is used to calculate the UAV’s trajectory, while the UAV’s state, sent from the UAV simulator to the VR Visualiser, is used to update the visual representation of the UAV. Figure 8 depicts this and shows the software tools used in the implementation of the architecture: MATLAB/Simulink^®^ for the UAV simulation, Unity3D to recreate the virtual home environment and Mosquitto as the open source MQTT broker selected.

As mentioned in Section 3, the UAV simulator described in [16] has been extended to include the different options for the relative monitoring altitude, the monitoring velocity and the monitoring radius. The virtual environment in which the action took place was a living room with a sofa in the centre and a TV in front of it, as can be seen on the right side of Figure 8. The avatar was sitting on the sofa and the UAV’s base station was located behind it. This configuration coincides with that described in the results of the simulations presented in Section 3.5. The task that the participants had to perform was simple; they only had to sit on the couch and watch TV. The UAV would then carry out various monitoring processes using the different variables considered. The virtual drone included a positional audio source that generated 3D spatial audio. The selected audio clip corresponded to the one produced by a real UAV during its flight, and was played in a loop during the monitoring process.

### 4.1. Procedure

Unlike our previous work described in [16], and due to the restrictions imposed by the COVID-19 pandemic regarding the use of head mounted equipment covering the face of the users, three videos were created showcasing the different alternatives considered for relative monitoring altitude, monitoring velocity and monitoring radius. Thus, the participants did not immerse themselves in the virtual environment but watched a video recorded from the perspective of an avatar sitting on the couch while watching TV inside the designed virtual living room.

Two different studies were carried out, first with descendants of physically impaired elderly people, and, after that, the experiment was repeated with elderly people. The participants received an email with a short description of the study and a link to an online questionnaire including the videos and some questions about it. The questionnaire was online from 1 May to 10 December 2020. It took an average time of 14 min to be filled by the participants in the evaluation.

### 4.2. Questionnaire

A questionnaire was designed using Microsoft Forms. It was divided into two main parts, a demographic questionnaire and a questionnaire about their preferences on the variables used in the study. This second questionnaire was designed specifically for this work, inspired by other approaches [38,39,40]. It was divided into three parts, one per each of the variables used in the study. For each part, a video was prepared showing each of the options per variable (e.g., three different flight heights) and then the participants had to answer to some questions regarding what they saw in the video and their preferences. The reason why the questionnaire was divided into three parts is that this would allow the participants to concentrate on only one parameter without considering the influence of the others. There was a total amount of 36 questions which were divided into preference questions, perceived safety, perceived supervision level, estimated distraction caused by the UAV flight and adequacy. A 5-point Likert scale ranging from strongly disagree to strongly agree was used to measure the responses.

### 4.3. Participants and Data Collection

For the first experiment, over 100 emails were sent to recruit participants. The potential participants were descendants of physically impaired elderly people who regularly attended a socio-cultural centre for older adults. We believed that the sons and daughters of these elderly people would be able to empathise with the problem of their ancestors and carry out the proposed experiment with great interest. A total amount of 37 questionnaires were received. From them, 13 participants were female (35%) and 24 male (65%), while the total mean age was M=41.41, SD=16.19, Max=75 and Min=22. Regarding their use of technology, 70% of the participants stated they are advanced users, 25% are basic users and 5% prefer not to use technology in their daily lives. As 70% of the participants were advanced users of technology, their responses to the questionnaire may probably have biased the results positively. However, the tendency is that more and more people get accustomed to this kind of equipment.

The experiment was repeated, this time with elderly people instead of their relatives. We performed the experiment with participants in the socio-cultural centre for older adults. We managed to obtain data from 23 participants (52% female and 48% male), with a mean age of M=74.48, SD=6.01, Max=87 and Min=66. This time, 35% of the participants stated that they prefer not to use technology, 39% were basic users and 26% were advanced users.

### 4.4. Data Analysis

The data gathered through the questionnaires are described using descriptive statistics. They include measures of central tendency (mean and median) and dispersion (standard deviation, SD, and interquartile range, IQR) as well as percentages of responses in a given range of answers. In the 5-point Likert scale used, the responses in the range 4–5 (agree or strongly agree) have been considered positive, 3 has been considered neutral, and 1–2 have been considered negative (disagree or strongly disagree). The distribution of responses for the questionnaire is plotted as stacked horizontal histograms, and pie charts are used to show the preference of the users about the different alternatives on relative monitoring altitude, monitoring velocity and monitoring radius. Since the data collected did not meet the requirements for normality (according to the Shapiro–Wilk test), the non-parametric Kruskal–Wallis test was used for null hypothesis testing in the comparison of different alternatives with a significance of 95%. In the cases in which the test found differences, the differences were studied using the Dunn’s post-hoc test together with a Bonferroni correction for pair-wise comparisons. IBM SPSS Statistics (version 24) and Microsoft Excel were used to conduct the statistical analyses.

## 5. Results

This section presents the results of both experiments. The results of the initial experiment with younger adults are provided in Section 5.1, while the results of the second one with older adults can be found in Section 5.2.

### 5.1. Results of the Experiment with Younger Adults (Relatives)

**Figure 9:**
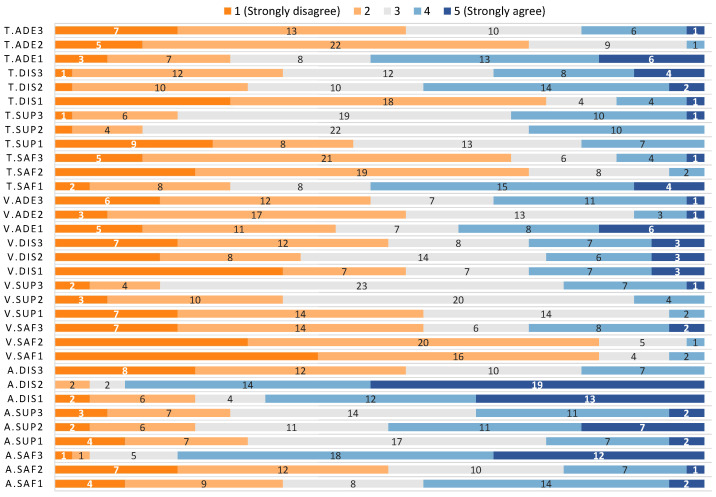
Distribution of responses for each question for the experiment with younger adults.

**Figure 10:**
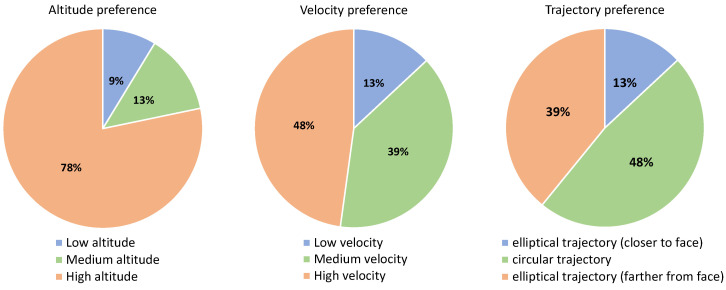
User preference for each of the variables measured for the experiment with younger adults.

The results of the first experiment are summarised in Table 5, as well as Figure 9 and Figure 10. Figure 10 uses pie charts to plot the preferences of the users regarding the variables under study. The preferred relative monitoring altitude is the highest one, which was selected by 78% of the participants (14% for low and 8% for medium). The difference in the preference for the monitoring velocity is not that big; 46% of the users selected high, 38% medium and 16% low. Finally, 54% of the participants selected the circular trajectory as their preferred one, followed by the elliptical trajectory when it is farther from their face (30%) and closer to their face (16%). Thus, the preferences are high altitude, high velocity and circular trajectory.

#### 5.1.1. Results for Relative Monitoring Altitude with Younger Adults

The results for the safety, supervision, and distraction questions are described in Table 5 and depicted in Figure 9. The results for safety show that users feel safer with the highest altitude, being the median value for this response 4 (IQR=1.00) and having 81% of the responses in the rage 4–5 (agree or strongly agree). This is in contrast with the medium (median value 2) and low (median value 3) altitudes. It is worth noting that the medium altitude had 51% of the responses in the range 1–2 (disagree or strongly disagree). This result can be observed in Figure 9, being much more blue in A.Saf3. The Kruskal–Wallis test shows a significant difference in the results obtained for each different altitude ($\chi_{\left(2\right)}^{2}$ = 31.20, *p* < 0.001), a post-hoc pairwise comparison revealed that this difference was between the highest altitude and the medium (*p* < 0.001) and the lower (*p* = 0.001), being the highest altitude perceived as safer.

Regarding supervision, the median values obtained for the three different altitudes is 3, only differing in the IQR value: IQR=2.00 for the highest altitude, and IQR=1.00 for the medium and lower altitudes. A deeper look at the results shows that medium and high altitudes had similar percentages of positive answers (35% for high, 49% for medium and 24% for low) while all had similar percentages of negative answers (27%, 22% and 30%, respectively for high, medium and low). The Kruskal–Wallis test did not show any difference in the results for the three options ($\chi_{\left(2\right)}^{2}$ = 4.29, *p* = 0.117).

Finally, for distraction, the median values were 2 (IQR=1.00), 5 (IQR=1.00) and 3 (IQR=2.00) for high, medium and low altitudes respectively. The percentages of positive answers for each altitude (high, medium, low) were 19%, 89% and 68%, while the negative ones were 54%, 5% and 22%. This difference can be noticed with a quick look at the A.Dis section of Figure 9. The Kruskal–Wallis test found a difference in the values gathered for each altitude ($\chi_{\left(2\right)}^{2}$ = 41.92, *p* < 0.001), being this difference between the high altitude and the other two (*p* < 0.001 for both), and the distraction lower for the high altitude.

#### 5.1.2. Results for Monitoring Velocity with Younger Adults

The medians of the results for safety for the different monitoring velocities were 4 (IQR=2.00), 4 (IQR=1.00) and 4 (IQR=1.00) for high, medium and low, respectively. The percentages of positive answers were 57%, 84% and 84%, while the percentages of negative answers were 27%, 3% and 5%. There was a significant difference in the data ($\chi_{\left(2\right)}^{2}$ = 9.97, *p* = 0.007) and a post-hoc test showed that this difference was between high and low velocity (*p* = 0.009), stating that the users considered the lower velocity safer.

The medians for the supervision data gathered for high, medium and low velocity were 3 (IQR=0.00), 3 (IQR=1.00) and 1 (IQR=1.00), respectively. The percentages of positive answers were 16%, 35% and 57%. The percentages of negative answers were 22%, 11% and 5%. The differences in the data are also apparent in Figure 9.

Regarding distraction, the median values were 4 (IQR=2.00) for high velocity, 3 (IQR=1.00) for medium velocity and 4 (IQR=3.00) for low velocity. The percentages of positive answers were 51%, 38% and 54% and the percentages of negative answers were 27%, 24% and 27%, respectively. In this case, the data look similar in Figure 9, and there were no statistically significant differences between the responses of the three groups ($\chi_{\left(2\right)}^{2}$ = 1.50, *p* = 0.473).

The median values obtained for the adequacy questions for velocity were 3 (IQR=2.00) for high, 4 (IQR=1.00) for medium and 3 (IQR=2.00) for low velocity. The percentages of positive answers were 49%, 54% and 43%, while the negative ones were 32%, 11% and 38%, respectively. One more time, there were no statistically significant differences between the responses of the three groups ($\chi_{\left(2\right)}^{2}$ = 2.25, *p* = 0.324).

#### 5.1.3. Results for Monitoring Radius with Younger Adults

The last group of questions was about the monitoring radius followed by the UAV during the monitoring process. The median values obtained for safety were 4 (IQR=1.00) for the elliptical trajectory farther from the user’s face, 4 (IQR=1.00) for the circular trajectory and 2 (IQR=2.00) for the elliptical trajectory closer to the user’s face. The percentages of positive answers were 70%, 73% and 27%, while they were 14%, 5% and 51% for the negative answers, respectively. The difference is noticeable this time with a quick look at the T.Saf block in Figure 9, and it was confirmed by the Kruskal–Wallis test ($\chi_{\left(2\right)}^{2}$ = 23.86, *p* < 0.001). The post-hoc test revealed that the difference was between the elliptical trajectory closer to the user’s face and both the circular (*p* < 0.001) and the elliptical trajectory farther from the user’s face (*p* < 0.001), being the perceived safety lower for the trajectory closer to the user’s face.

The medians for supervision were 3 (IQR=1.00) for the elliptical trajectory farther from the user’s face, 3 (IQR=1.00) for the circular trajectory and 3 (IQR=1.00) for the elliptical trajectory closer to the user’s face. The percentages of positive answers were 19%, 14% and 46%, while they were 30%, 27% and 19% for the negative answers, respectively. There was a significant difference ($\chi_{\left(2\right)}^{2}$ = 8.78, *p* < 0.01) between the elliptical trajectory closer to the user’s face and the circular trajectory (*p* = 0.028) and the elliptical trajectory farther from the user’s face (*p* = 0.034). The post-hoc test states that the perceived level of supervision is higher for the trajectory closer to the user’s face.

About distraction, the median values for monitoring radius were 3 (IQR=2.00) for the elliptical trajectory farther from the user’s face, 3 (IQR=2.00) for the circular trajectory and 4 (IQR=1.00) for the elliptical trajectory closer to the user’s face. The percentages of positive answers were 35%, 30% and 76%, while they were 32%, 43% and 14% for the negative answers, respectively. There was a significant difference in the responses ($\chi_{\left(2\right)}^{2}$ = 20.15, *p* < 0.001), being the level of distraction higher for the trajectory closer to the user’s face as compared to the circular trajectory (*p* = 0.001) and the elliptical trajectory farther from the user’s face (*p* < 0.001).

Finally, the median values regarding adequacy were 4 (IQR=1.00), 4 (IQR=1.00) and 2 (IQR=2.00). The percentages of positive answers were 54%, 73% and 27%, while they were 19%, 3% and 51% for the negative answers, respectively. The Kruskal–Wallis test found a difference in the values gathered for each monitoring radius ($\chi_{\left(2\right)}^{2}$ = 25.98, *p* < 0.001), being this difference between the elliptical trajectory closer to the user’s face and the other two (*p* < 0.001 for the circular trajectory and *p* < 0.001 for elliptical trajectory farther from the user’s face), being the adequacy lower for the trajectory closer to the user’s face.

### 5.2. Results of the Experiment with Older Adults (with Physical Impairments)

**Figure 11:**
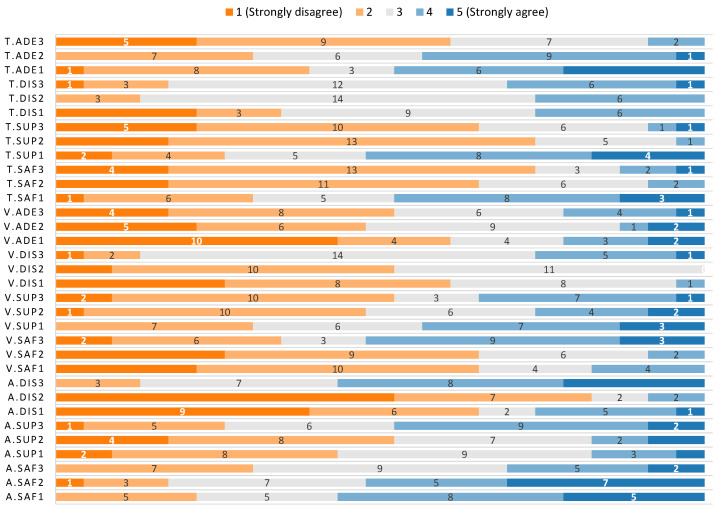
Distribution of responses for each question for older adults.

**Figure 12:**
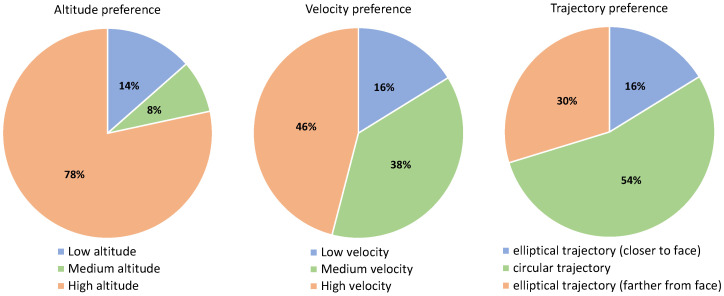
User preference for each of the variables measured for older adults.

This section presents the results of the second experiment, which are summarised in Table 6, Figure 11 and Figure 12. The results for preferences regarding the three variables under study are similar to the first study with younger adults. Regarding relative monitoring altitude, 78% preferred the highest altitude, 13% the medium and 9% the lowest altitude. Again, the difference in the preferences is not that big for the monitoring velocity: 48%, 39% and 13% for high, medium and low velocity, respectively. Regarding the monitoring trajectory, the preference of the participants was 48% for circular, 39% for the elliptical trajectory when it is farther from their face, and 13% when it is closer to their face.

#### 5.2.1. Results for Relative Monitoring Altitude with Older Adults

Regarding safety for relative monitoring altitude, the results show that the participants did not feel positive about any of the options; the percentages of positive (5 or 4) vs. negative (1 or 2) answers are 22%, 17% and 30% for low, medium and high altitudes for positive answers, while for the negative ones are 57%, 52% and 30%, respectively. Therefore, the participants showed a neutral attitude towards the high altitude only (39% of the answers). For the other two alternatives, the trend in the responses is towards neutral or negative attitude. The median values of the data support this, as it was 2 for low and medium altitude (IQR=1.00 and 2.00 respectively), and 3 (IQR=2.00) for the high altitude. Despite this, the Kruskal–Wallis test fails at finding a significant difference in the results obtained for each different altitude ($\chi_{\left(2\right)}^{2}$ = 3.50, p=0.174).

For supervision, the median values for the three different altitudes are similar to those obtained for younger adults (3 with IQR=2.00 for low, 4 with IQR=1.00 for medium and 3 with IQR=1.50 for high altitude). The Kruskal–Wallis test did not show any difference in the results for the three options ($\chi_{\left(2\right)}^{2}$ = 5.71, p=0.058). Despite this, a closer look at the data shows that the percentages of positive and negative answers are 43% for positive ones and 17% for negative answers in the case of low altitude, 52% and 17% for medium altitude and 26% and 48% for high altitude. This is aligned with the results obtained for altitude preference, as older adults selected high as the better altitude.

The median values obtained for distraction were 2 (IQR=1.00), 5 (IQR=1.00) and 4 (IQR=2.50) for high, medium and low altitudes, respectively, which are similar to the results obtained for younger adults. The percentages of positive and negative answers for each altitude (high, medium, low) are also similar to those obtained for younger adults: 13%, 83% and 65% for positive answers and 57%, 9% and 26% for negative answers. There is a statistically significant difference in the values gathered for each altitude according to the Kruskal–Wallis test ($\chi_{\left(2\right)}^{2}$ = 24.55, p<0.001), with differences between the high altitude and the other two (p=0.001 for the difference with low altitude and p<0.001 for the difference with medium altitude), being the distraction lower for the high altitude.

#### 5.2.2. Results for Monitoring Velocity with Older Adults

The different monitoring velocities had median values for safety of 2 (IQR=2.00), 4 (IQR=1.50) and 4 (IQR=1.00) for high, medium and low, respectively. 35%, 65% and 65% were positive responses, while 52%, 9% and 17% were negative answers. A statistically significant difference was found in the data ($\chi_{\left(2\right)}^{2}$ = 9.76, *p* = 0.008). This difference was between high and low velocity (*p* = 0.036) and between high and medium velocity (*p* = 0.012). Thus, the older adults considered the lower and medium velocities safer.

The medians for the supervision data gathered for high, medium and low velocity were 4 (IQR=2.00), 3 (IQR=1.50) and 3 (IQR=2.00), respectively. The percentages of positive answers were 52%, 48% and 30%, and 35%, 26% and 43% for the negative answers. Again, and similarly to the data obtained for younger adults, the apparent differences in the data are not significant enough according to the Kruskal–Wallis test ($\chi_{\left(2\right)}^{2}$ = 2.70, *p* = 0.259).

The case of distraction was different. The median values were 3 (IQR=0.50) for high velocity, 4 (IQR=1.00) for medium velocity and 4 (IQR=1.50) for low velocity. The percentages of positive answers were 13%, 52% and 61% and the percentages of negative answers were 26%, 0% and 4%, respectively. There was a significant difference between them ($\chi_{\left(2\right)}^{2}$ = 15.18, *p* = 0.001). The difference was between the low and high velocities (*p* = 0.001), and medium and high velocities (*p* = 0.009), being the level of perceived distraction lower for the high velocity.

The last variable measured for monitoring velocity was adequacy, and the results obtained were 4 (IQR=1.00) for high, 3 (IQR=1.00) for medium and 4 (IQR=2.00) for low velocity. The percentages of positive answers were 52%, 48% and 61%, while the negative ones were 22%, 13% and 22%, respectively. No significant differences could be found between the responses of the three groups ($\chi_{\left(2\right)}^{2}$ = 1.35, *p* = 0.510).

#### 5.2.3. Results for Monitoring Radius with Older Adults

Regarding safety, the median values for safety were 4 (IQR=0.50) for the elliptical trajectory farther from the user’s face, 4 (IQR=1.00) for the circular trajectory and 3 (IQR=2.00) for the elliptical trajectory closer to the user’s face. The percentages of positive answers were 74%, 65% and 30%, while the negative ones were 13%, 9% and 48%, respectively. The difference was confirmed by the Kruskal–Wallis test ($\chi_{\left(2\right)}^{2}$ = 11.99, *p* = 0.002). This difference was between the elliptical trajectory closer to the user’s face and both the circular (*p* = 0.011) and the elliptical trajectory farther from the user’s face (*p* = 0.006), being the perceived safety lower for the trajectory closer to the user’s face.

The medians for supervision were 4 (IQR=1.00) for the elliptical trajectory farther from the user’s face, 4 (IQR=0.50) for the circular trajectory and 2 (IQR=1.50) for the elliptical trajectory closer to the user’s face. The percentages of positive answers were 65%, 74% and 26%, while they were 9%, 4% and 52% for the negative answers, respectively. The Kruskal–Wallis test found a significant difference ($\chi_{\left(2\right)}^{2}$ = 14.27, *p* = 0.001). The post-hoc test revealed that this difference was between the elliptical trajectory closer to the user’s face and both the circular trajectory (*p* = 0.002) and the elliptical trajectory farther from the user’s face (*p* = 0.006). In both cases, the perceived level of supervision is higher for the trajectory closer to the user’s face.

No significant difference could be found for distraction ($\chi_{\left(2\right)}^{2}$ = 1.93, *p* < 0.381). The median values were similar for the different monitoring radius: 3 (IQR=1.00) for the elliptical trajectory farther from the user’s face, 3 (IQR=0.50) for the circular trajectory and 3 (IQR=1.50) for the elliptical trajectory closer to the user’s face. The same result was found for the percentages of positive answers, which were 17%, 13% and 35% for positive responses and 30%, 26% and 26% for the negative ones, respectively.

Adequacy was the last variable, and the median values obtained were 4 (IQR=1.00), 3 (IQR=2.00) and 3 (IQR=2.00) for the trajectories farther from the user’s face, circular and closer to the user’s face, respectively. The percentages of positive answers were 61%, 30% and 39%, while they were 9%, 43% and 48% for the negative answers, respectively. This time, a significant difference could be found for each monitoring radius ($\chi_{\left(2\right)}^{2}$ = 10.17, *p* = 0.006). The post-hoc test found that this difference was between the elliptical trajectory farther from the user’s face and the other two (*p* = 0.019 for the circular trajectory and *p* = 0.016 for elliptical trajectory closer to the user’s face). In both cases, the adequacy is higher for the trajectory farther from the user’s face.

## 6. Discussion

This paper belongs to a research line in socially assistive UAVs with potential for dependent people, including ageing adults. The paper has presented an assistive UAV whose mission is to perform a monitoring flight from time to time to determine a person’s condition and a possible assistance. This monitoring flight basically consists of a series of manoeuvres to take-off, get close to the person, fly around the person to obtain facial images and then return to its base. Moreover, a survey was conducted to evaluate the users’ sense of safety and comfort in a VR home environment.

The 60 participants (37 descendants and 23 older adults) evaluated several parameters of the UAV’s trajectory during the monitoring process. The main aim of this evaluation was to study the impact of different alternatives for three key parameters of the monitoring process of an assistive UAV: the relative monitoring altitude, the monitoring velocity and the monitoring radius. For each parameter, three alternatives were implemented in our simulation platform and tested using a VR environment. The user preferences were consistent with the answers they provided about questions regarding the perceived safety, supervision, distraction and adequacy. Table 7 summarises the main results presented in the previous section, which are used in this discussion.

High altitude was selected as the most appropriate altitude for the UAV monitoring process by most participants. At the same time, it was perceived as the safest one, especially for younger adults, followed by the lower altitude.A possible explanation is that the participants felt that a higher altitude would avoid collisions with the UAV, which could be dangerous specially at the user head level (medium altitude). Even though there is no statistically significant difference in the perceived safety for older adults, there is a reduction in the results obtained for them (means and percentages) when compared to younger adults. This reduction is more noticeable in the higher altitude (81% of positive answers for younger adults and 30% for older adults), where most of the responses were neutral (40%). Hence, older adults may not feel completely safe with this monitoring altitude, but, more importantly, they do not feel in danger, so it is very likely that they will tolerate and accept it. Regarding the perceived supervision level, no significant differences could be found, despite being the medium altitude perceived slightly more negative than the other two. For distraction, the highest altitude was perceived to cause the lowest level of distraction in a statistically significant manner, probably because it was far from their line of sight. Therefore, it seems reasonable to think that the highest altitude is the best one for monitoring processes.

High velocity was considered as the most appropriate one for monitoring, followed by medium velocity. However, it was perceived as less safe and with higher level of perceived supervision in younger adults than the low velocity. That was not exactly the case for distraction, since no statistically significant difference was found in the data for younger adults. Notice, however, that a lower velocity distracts more than high velocity in older adults. Regarding the adequacy of the monitoring velocities, the data gathered was very similar for all of them, so, again, no statistically significant difference could be found. In this case, either the high or the medium velocity could be selected when designing the monitoring process of a UAV.

The circular trajectory was selected by most of the participants as their preferred one, while the elliptical trajectory passing closer to their face was the one they liked the least. This is in line with the results for safety and supervision, as closer to their face is perceived as less safe, with too much supervision and causing more distractions to them. For distraction, the same applies to younger adults, but no significant difference was detected for older adults. Moreover, there is no statistically significant difference between circular and elliptical trajectory passing farther from the user’s face, only a slightly higher perceived supervision for the circular one, which could be related to the fact that the trajectory passes closer to the user’s face. Unlike what was observed when comparing means and percentages of younger and older adults for the other two variables, perceived safety is not affected. Thus, according to the data gathered, the circular trajectory would be preferred over elliptical ones, but the elliptical trajectory passing farther from the user’s face could be also a good choice according to safety, supervision level and distraction.

The limitations of this evaluation are related to the difficulties of performing face-to-face experiments using VR facilities. Due to the current situation amid the COVID-19 pandemic, a series of videos were shown to the participants. The validation through videos, and not in an immersive VR environment, may have distorted some of the results. The authors, therefore, will hopefully repeat the experiments in a close future. In addition, the sample size must also be incremented to reinforce the current conclusions derived from the study.

Nonetheless, the authors believe that using VR as an alternative to physical prototyping saves time, provides a high flexibility and enables iterative testing possibilities in the design of socially assistive technologies incorporating different aspects of user trust in automation. Our future work also aims to make progress on the safety issues associated with flying a UAV at home, with the consequent risk of a domestic accident. Since all the instructions that come with current commercial UAVs advise against flying close to people, it is our intention to contact experts in miniaturisation in order to achieve the maximum physical integrity of the person.

## Figures and Tables

**Table 1:** Trajectory planner’s states for the monitoring process. (Part I—States from 0 to 6). Reference trajectories for the position (x,y,z) and yaw angle (ψ) of a quadrotor UAV monitoring a person whose position $\left(x_{p},y_{p},z_{p}\right)$ and orientation ($\alpha_{p})\;$are known.

State	Reference Trajectories	Parameters and Condition [C]
0	$x_{0}^{*}\left(t\right)=x_{b}$	$\left(x_{b},y_{b},z_{b}\right)$: base position [m]
	$y_{0}^{*}\left(t\right)=y_{b}$	ψ(0): initial yaw angle (by default 0) [rad]
	$z_{0}^{*}\left(t\right)=z_{b}$
	$\psi_{0}^{*}\left(t\right)=\psi \left(0\right)$
		[C] If Instruction is Received → State 1
1	$x_{1}^{*}\left(t\right)=x_{i1}$	$z_{r}$: relative monitoring altitude [m]
	$y_{1}^{*}\left(t\right)=y_{i1}$	$v_{z}$: velocity in Z-Axis [m/s]
	$z_{1}^{*}\left(t\right)=z_{i1}+\left(\frac{t-t_{i1}}{t_{f1}-t_{i1}}\right)\cdot \left(z_{m}-z_{i1}\right)$
	$\psi_{1}^{*}\left(t\right)=\psi_{i1}$
	where: $t_{f1}=t_{i1}+\left|\frac{z_{m}-z_{i1}}{v_{z}}\right|$; $z_{m}=z_{p}+z_{r}$
		[C] If $\left(x,y,z\right)=\left(x_{b},y_{b},z_{m}\right)\to$ State 2
2	$x_{2}^{*}\left(t\right)=x_{i2}$	$\omega_{\psi }$: angular velocity (yaw) [rad/s]
	$y_{2}^{*}\left(t\right)=y_{i2}$	$\alpha_{camera}$: camera’s angle [rad]
	$z_{2}^{*}\left(t\right)=z_{i2}$
	$\psi_{2}^{*}\left(t\right)=\psi_{i2}+\left(\frac{t-t_{i2}}{t_{f2}-t_{i2}}\right)\cdot \left(\psi_{f2}-\psi_{i2}\right)$
	where: $t_{f2}=t_{i2}+\left|\frac{\psi_{f2}-\psi_{i2}}{\omega_{\psi }}\right|$;
	$\psi_{f2}=\left(\alpha -\alpha_{camera}\right);\;\alpha =arctan\left(\frac{y_{p}-y_{i2}}{x_{p}-x_{i2}}\right)$
		[C] If $\psi =\psi_{f2}=\left(\alpha -\alpha_{camera}\right)\to$ State 3
3	$x_{3}^{*}\left(t\right)=x_{i3}+v_{d}\cdot \frac{\left(x_{sp}-x_{i3}\right)}{d}\cdot \left(t-t_{i3}\right)$	$R_{x}$: radius on the X-axis [m]
	$y_{3}^{*}\left(t\right)=y_{i3}+v_{d}\cdot \frac{\left(y_{sp}-y_{i3}\right)}{d}\cdot \left(t-t_{i3}\right)$	$R_{y}$: radius on the Y-axis [m]
	$z_{3}^{*}\left(t\right)=z_{i3}$	$v_{d}$: diagonal velocity [m/s]
	$\psi_{3}^{*}\left(t\right)=\psi_{i3}$
	where: $d=\sqrt{{\left(x_{sp}-x_{i3}\right)}^{2}+{\left(y_{sp}-y_{i3}\right)}^{2}};$
	$(x_{sp},y_{sp})\Rightarrow$ see Section 3.2
		[C] If $\left(x,y,z\right)=\left(x_{sp},y_{sp},z_{sp}\right)\to$ State 4
4	$x_{4}^{*}\left(t\right)=x_{i4}$	$t_{s4}$: timer [s]
	$y_{4}^{*}\left(t\right)=y_{i4}$	
	$z_{4}^{*}\left(t\right)=z_{i4}$
	$\psi_{4}^{*}\left(t\right)=\psi_{i4}$
		[C] If $t_{i4}+t_{s4}\to$ State 5
5	$x_{5}^{*}\left(t\right)=x_{p}+R_{x}\cos\left(\gamma \right)\cos\left(\alpha_{p}\right)-R_{y}\sin\left(\gamma \right)\sin\left(\alpha_{p}\right)$	$R_{x}$: radius on the X-axis [m]
	$y_{5}^{*}\left(t\right)=y_{p}+R_{x}\cos\left(\gamma \right)\sin\left(\alpha_{p}\right)+R_{y}\sin\left(\gamma \right)\cos\left(\alpha_{p}\right)$	$R_{y}$: radius on the Y-axis [m]
	$z_{5}^{*}\left(t\right)=z_{i5}$	$\omega_{m}$: monitoring angular velocity [rad/s]
	$\psi_{5}^{*}\left(t\right)=arctan\left(\frac{y_{p}-y_{5}^{*}\left(t\right)}{x_{p}-x_{5}^{*}\left(t\right)}\right)-\alpha_{camera}$	$\alpha_{camera}$: camera’s angle [rad]
	where: $\gamma =\gamma_{sp}+\omega_{m}\cdot \left(t-t_{i5}\right);$
	$\gamma_{sp}\Rightarrow$ see Section 3.3
	$\omega_{m}\Rightarrow$ see Table 3	[C] If $\left(x,y,z\right)=\left(x_{ph},y_{ph},z_{ph}\right)\to$ State 6
6	$x_{6}^{*}\left(t\right)=x_{i6}$	$t_{s6}$: timer [s]
	$y_{6}^{*}\left(t\right)=y_{i6}$	
	$z_{6}^{*}\left(t\right)=z_{i6}$
	$\psi_{6}^{*}\left(t\right)=\psi_{i6}$
		[C] If $t_{i6}+t_{s6}\to$ State 7

Notation ⇒$\left(x_{n}^{*}\left(t\right),y_{n}^{*}\left(t\right),z_{n}^{*}\left(t\right)\right)$: reference position in state *n*; $\psi_{n}^{*}\left(t\right)$ reference yaw angle in state *n*; $t_{in}$: initial time of state *n*; $t_{fn}$: final time of state *n*; $x_{in}=x\left(t_{in}\right)$: initial value of *x* coordinate at the beginning of state *n* (at instant $t_{in}$); $y_{in}=y\left(t_{in}\right)$: initial value of *y* coordinate at the beginning of state *y* (at instant $t_{in}$); $z_{in}=z\left(t_{in}\right)$: initial value of *z* coordinate at the beginning of state *n* (at instant $t_{in}$); $\psi_{in}=\psi \left(t_{in}\right)$: initial value of ψ angle at the beginning of state *n* (at instant $t_{in}$).

**Table 2:** Trajectory planner’s states for the monitoring process. (Part II—States from 7 to 11). Reference trajectories for the position (x,y,z) and yaw angle (ψ) of a quadrotor UAV monitoring a person whose position $\left(x_{p},y_{p},z_{p}\right)$ and orientation ($\alpha_{p})\;$are known.

State	Reference Trajectories	Parameters and Condition [C]
7	$x_{7}^{*}\left(t\right)=x_{p}+R_{x}\cos\left(\gamma \right)\cos\left(\alpha_{p}\right)-R_{y}\sin\left(\gamma \right)\sin\left(\alpha_{p}\right)$	$R_{x}$: radius on the X-axis [m]
	$y_{7}^{*}\left(t\right)=y_{p}+R_{x}\cos\left(\gamma \right)\sin\left(\alpha_{p}\right)+R_{y}\sin\left(\gamma \right)\cos\left(\alpha_{p}\right)$	$R_{y}$: radius on the Y-axis [m]
	$z_{7}^{*}\left(t\right)=z_{i7}$	$\omega_{m}$: monitoring angular velocity [rad/s]
	$\psi_{7}^{*}\left(t\right)=arctan\left(\frac{y_{p}-y_{7}^{*}\left(t\right)}{x_{p}-x_{7}^{*}\left(t\right)}\right)-\alpha_{camera}$	$\alpha_{camera}$: camera’s angle [rad]
	where: $\gamma =\gamma_{ph}+\omega_{m}\cdot \left(t-t_{i7}\right);$ $\gamma_{ph}=0$	
	$\omega_{m}\Rightarrow$ see Table 3	[C] If $\left(x,y,z\right)=\left(x_{sp},y_{sp},z_{sp}\right)\to$ State 8
8	$x_{8}^{*}\left(t\right)=x_{i8}$	$\omega_{\psi }$: angular velocity (yaw) [rad/s]
	$y_{8}^{*}\left(t\right)=y_{i8}$	
	$z_{8}^{*}\left(t\right)=z_{i8}$	
	$\psi_{8}^{*}\left(t\right)=\psi_{i8}+\left(\frac{t-t_{i8}}{t_{f8}-t_{i8}}\right)\cdot \left(\psi_{f8}-\psi_{i8}\right)$	
	where: $t_{f8}=t_{i8}+\left|\frac{\psi_{f8}-\psi_{i8}}{\omega_{\psi }}\right|$; $\psi_{f8}=\left(\psi_{i8}-\pi \right)$	
		[C] If $\psi =\psi_{f8}=\left(\psi_{i8}-\pi \right)\to$ State 9
9	$x_{9}^{*}\left(t\right)=x_{i9}+v_{d}\cdot \frac{\left(x_{b}-x_{i9}\right)}{d}\cdot \left(t-t_{i9}\right)$	$\left(x_{b},y_{b},z_{b}\right)$: base position [m]
	$y_{9}^{*}\left(t\right)=y_{i9}+v_{d}\cdot \frac{\left(y_{b}-y_{i9}\right)}{d}\cdot \left(t-t_{i9}\right)$	$v_{d}$: diagonal velocity [m/s]
	$z_{9}^{*}\left(t\right)=z_{i9}$	
	$\psi_{9}^{*}\left(t\right)=\psi_{i9}$	
	where: $d=\sqrt{{\left(x_{b}-x_{i9}\right)}^{2}+{\left(y_{b}-y_{i9}\right)}^{2}}$	
		[C] If $\left(x,y,z\right)=\left(x_{b},y_{b},z_{m}\right)\to$ State 10
10	$x_{10}^{*}\left(t\right)=x_{i10}$	$\omega_{\psi }$: angular velocity (yaw) [rad/s]
	$y_{10}^{*}\left(t\right)=y_{i10}$	$\psi_{0}$: initial yaw angle (by default 0) [rad]
	$z_{10}^{*}\left(t\right)=z_{i10}$	
	$\psi_{10}^{*}\left(t\right)=\psi_{i10}+\left(\frac{t-t_{i10}}{t_{f10}-t_{i10}}\right)\cdot \left(\psi \left(0\right)-\psi_{i10}\right)$	
	where: $t_{f10}=t_{i10}+\left|\frac{\psi \left(0\right)-\psi_{i10}}{\omega_{\psi }}\right|$	
		[C] If ψ=ψ(0)→ State 11
11	$x_{11}^{*}\left(t\right)=x_{i11}$	$\left(x_{b},y_{b},z_{b}\right)$: base position [m]
	$y_{11}^{*}\left(t\right)=y_{i11}$	$v_{z}$: velocity in Z-Axis [m/s]
	$z_{11}^{*}\left(t\right)=z_{i11}+\left(\frac{t-t_{i11}}{t_{f11}-t_{i11}}\right)\cdot \left(z_{b}-z_{i11}\right)$	
	$\psi_{11}^{*}\left(t\right)=\psi_{i11}$	
	where: $t_{f11}=t_{i11}+\left|\frac{z_{b}-z_{i11}}{v_{z}}\right|$
		[C] If $\left(x,y,z\right)=\left(x_{b},y_{b},z_{b}\right)\to$ State 0

Notation ⇒$\left(x_{n}^{*}\left(t\right),y_{n}^{*}\left(t\right),z_{n}^{*}\left(t\right)\right)$: reference position in state *n*; $\psi_{n}^{*}\left(t\right)$ reference yaw angle in state *n*; $t_{in}$: initial time of state *n*; $t_{fn}$: final time of state *n*; $x_{in}=x\left(t_{in}\right)$: initial value of *x* coordinate at the beginning of state *n* (at instant $t_{in}$); $y_{in}=y\left(t_{in}\right)$: initial value of *y* coordinate at the beginning of state *y* (at instant $t_{in}$); $z_{in}=z\left(t_{in}\right)$: initial value of *z* coordinate at the beginning of state *n* (at instant $t_{in}$); $\psi_{in}=\psi \left(t_{in}\right)$: initial value of ψ angle at the beginning of state *n* (at instant $t_{in}$).

**Table 3:** Values established in the simulation tests for each of the parameters under study; relative monitoring altitude, monitoring velocity, and monitoring radius (type/shape of trajectory).

Parameter	1st Value (Extreme 1)	2nd Value (Intermediate)	3rd Value (Extreme 2)
Relative Monitoring Altitude	Low/Below	Medium/Centred	High/Above
$z_{r}\Rightarrow z_{m}=z_{p}+z_{r}$	$z_{r}=-0.30$ [m]	$z_{r}=0$ [m]	$z_{r}=+0.30$ [m]
Monitoring Velocity	Low Velocity	Medium Velocity	High Velocity
$\omega_{m}=\omega_{\gamma }\cdot f_{\omega }$	$f_{\omega }=0.65$	$f_{\omega }=1$	$f_{\omega }=1.35$
Monitoring Radius	Closer to the Face	Equidistant (Circular)	Farther from the Face
(Ellipse) $R_{x}$	$R_{x}=0.90$ [m]	$R_{x}=1.30$ [m]	$R_{x}=1.70$ [m]
(Ellipse) $R_{y}$	$R_{y}=1.70$ [m]	$R_{y}=1.30$ [m]	$R_{y}=0.90$ [m]

**Table 4:** Parameters defined in the UAV simulator (MATLAB/Simulink^®^ environment).

Description	Value	Units
Simulation		
Sample Time	Ts=0.01	[s]
Simulation Time	t=250	[s]
Quadrotor UAV		
Initial Position (Base Position)	$\left(x_{b},y_{b},z_{b}\right)=\left(0,0,0\right)$	[m]
Initial Yaw Angle	ψ(0)=0	[rad]
Camera’s Angle	$\alpha_{camera}=\pi /4$	[rad]
Mass	m=1	[Kg]
Trajectory Planner		
Fixed Parameters:		
Velocity in Z axis [state 1—landing/state 11—takeoff]	$v_{z}=\left[8.6\times 10^{-2}/4.3\times 10^{-2}\right]$	[m/s]
Velocity in XY Diagonal Motion [state 3/state 9]	$v_{d}=\left[0.1/0.2\right]$	[m/s]
Angular Velocity for Yaw Adjustment	$\omega_{\psi }=3\pi /50$	[rad/s]
Angular Velocity for Ellipsoidal Motion	$\omega_{\gamma }=9\pi /250$	[rad/s]
Timer of State 4—Waiting in Safety Position	$t_{s4}=5$	[s]
Timer of State 6—Data Capture	$t_{s6}=0$	[s]
Variable Parameters ⇒ see Table 3 and Figure 7		

**Table 5:** Questionnaire and participants’ responses for the experiment with younger adults.

Question	Mean	SD	Median	IQR
Altitude				
Safety				
A.Saf1: I felt safe during the monitoring process by the UAV flying below my head	3.03	1.14	3.00	2.00
A.Saf2: I felt safe during the monitoring process by the UAV flying in front of my head	2.54	1.10	2.00	1.00
A.Saf3: I felt safe during the monitoring process by the UAV flying above my head	4.05	0.91	4.00	1.00
Supervision				
A.Sup1: I feel that there is too much supervision by the UAV flying below my head	2.89	1.02	3.00	1.00
A.Sup2: I feel that there is too much supervision by the UAV flying in front of my head	3.41	1.14	3.00	2.00
A.Sup3: I feel that there is too much supervision by the UAV flying above my head	3.05	1.03	3.00	1.00
Distraction				
A.Dis1: I think the UAV would distract me from my daily routine by flying below my head	3.76	1.26	4.00	2.00
A.Dis2: I think the UAV would distract me from my daily routine by flying in front of my head	4.35	0.82	5.00	1.00
A.Dis3: I think the UAV would distract me from my daily routine by flying above my head	2.43	1.04	2.00	1.00
Velocity				
Safety				
V.Saf1: I felt safe during the monitoring process by the UAV flying at a low velocity	4.19	0.84	4.00	1.00
V.Saf2: I felt safe during the monitoring process by the UAV flying at a medium velocity	4.11	0.74	4.00	1.00
V.Saf3: I felt safe during the monitoring process by the UAV flying at a high velocity	3.43	1.19	4.00	2.00
Supervision				
V.Sup1: I feel that there is too much supervision by the UAV flying at a low velocity	3.70	0.85	4.00	1.00
V.Sup2: I feel that there is too much supervision by the UAV flying at a medium velocity	3.32	0.78	3.00	1.00
V.Sup3: I feel that there is too much supervision by the UAV flying at a high velocity	2.97	0.80	3.00	0.00
Distraction				
V.Dis1: I think the UAV would distract me from my daily routine by flying at a low velocity	3.54	1.37	4.00	3.00
V.Dis2: I think the UAV would distract me from my daily routine by flying at a medium velocity	3.22	1.16	3.00	1.00
V.Dis3: I think the UAV would distract me from my daily routine by flying at a high velocity	3.35	1.23	4.00	2.00
Adequacy				
V.Ade1: I found the low velocity of the UAV adequate	3.03	1.32	3.00	2.00
V.Ade2: I found the medium velocity of the UAV adequate	3.49	0.87	4.00	1.00
V.Ade3: I found the high velocity of the UAV adequate	3.30	1.15	3.00	2.00
Trajectory				
Safety				
T.Saf1: I felt safe during the monitoring process by the UAV flying elliptically (closer to face)	2.70	1.10	2.00	2.00
T.Saf2: I felt safe during the monitoring process by the UAV flying following a circular trajectory	3.89	0.81	4.00	1.00
T.Saf3: I felt safe during the monitoring process by the UAV flying elliptically (farther from face)	3.68	0.94	4.00	1.00
Supervision				
T.Sup1: I feel that there is too much supervision by the UAV flying elliptically (closer to face)	3.51	1.07	3.00	1.00
T.Sup2: I feel that there is too much supervision by the UAV flying following a circular trajectory	2.89	0.70	3.00	1.00
T.Sup3: I feel that there is too much supervision by the UAV flying elliptically (farther from face)	2.89	0.81	3.00	1.00
Distraction				
T.Dis1: I think the UAV would distract me from my daily routine by flying elliptically (closer to face)	3.86	1.03	4.00	1.00
T.Dis2: I think the UAV would distract me from my daily routine by flying following a circular trajectory	2.84	0.99	3.00	2.00
T.Dis3: I think the UAV would distract me from my daily routine by flying elliptically (farther from face)	2.95	1.05	3.00	2.00
Adequacy				
T.Ade1: I found the UAV’s monitoring distance to be correct by the UAV flying elliptically (closer to face)	2.68	1.20	2.00	2.00
T.Ade2: I found the UAV’s monitoring distance to be correct by the UAV flying following a circular trajectory	3.84	0.69	4.00	1.00
T.Ade3: I found the UAV’s monitoring distance to be correct by the UAV flying elliptically (farther from face)	3.51	1.07	4.00	1.00

**Table 6:** Questionnaire and participants’ responses for the second experiment with older adults.

Question	Mean	SD	Median	IQR
Altitude				
Safety				
A.Saf1	2.43	1.08	2.00	1.00
A.Saf2	2.39	1.20	2.00	2.00
A.Saf3	2.91	0.95	3.00	2.00
Supervision				
A.Sup1	3.30	0.97	3.00	1.00
A.Sup2	3.43	1.16	4.00	1.00
A.Sup3	2.74	1.05	3.00	1.50
Distraction				
A.Dis1	3.74	1.32	4.00	2.50
A.Dis2	4.26	0.96	5.00	1.00
A.Dis3	2.35	0.98	2.00	1.00
Velocity				
Safety				
V.Saf1	3.70	1.02	4.00	1.00
V.Saf2	3.83	0.94	4.00	1.50
V.Saf3	2.78	1.24	2.00	2.00
Supervision				
V.Sup1	2.74	1.05	3.00	2.00
V.Sup2	3.17	1.07	3.00	1.50
V.Sup3	3.22	1.13	4.00	2.00
Distraction				
V.Dis1	3.83	0.89	4.00	1.50
V.Dis2	3.61	0.66	4.00	1.00
V.Dis3	2.87	0.81	4.00	2.00
Adequacy				
V.Ade1	3.74	1.39	4.00	2.00
V.Ade2	3.48	1.16	3.00	1.00
V.Ade3	3.43	1.12	4.00	1.00
Trajectory				
Safety				
T.Saf1	2.74	1.14	3.00	2.00
T.Saf2	3.74	0.86	4.00	1.00
T.Saf3	3.74	1.01	4.00	0.50
Supervision				
T.Sup1	2.65	1.23	2.00	1.50
T.Sup2	3.87	0.76	4.00	0.50
T.Sup3	3.74	1.01	4.00	1.00
Distraction				
T.Dis1	3.30	1.11	3.00	1.50
T.Dis2	2.87	0.76	4.00	0.50
T.Dis3	2.87	0.87	3.00	1.00
Adequacy				
T.Ade1	2.74	1.29	3.00	2.00
T.Ade2	2.83	0.94	3.00	2.00
T.Ade3	3.74	0.92	4.00	1.00

**Table 7:** Summary of the results for younger and older adults.

		Younger Adults	Older Adults
Altitude			
	Preference	High altitude	High altitude
	Safety	High altitude safer than medium and low altitudes	No significant difference
	Supervision	No significant difference	No significant difference
	Distraction	Medium and low altitudes distract more than high altitude	Medium and low altitudes distract more than high altitude
Velocity			
	Preference	High velocity	High velocity
	Safety	Lower velocity safer than high velocity	Lower and medium velocities safer than high velocity
	Supervision	Low velocity has more perceived supervision than high velocity	No significant difference
	Distraction	No significant difference	Lower velocity distracts more than high velocity
	Adequacy	No significant difference	No significant difference
Trajectory			
	Preference	Circular trajectory	Circular trajectory
	Safety	Circular and elliptical trajectory farther from the user’s face safer than elliptical trajectory closer to the user’s face	Circular and elliptical trajectory farther from the user’s face safer than elliptical trajectory closer to the user’s face
	Supervision	Elliptical trajectory close to the user has more perceived supervision than circular and elliptical trajectory far from the user	Higher supervision for elliptical trajectory close to the user than circular and elliptical trajectory far from the user
	Distraction	Elliptical trajectory close to the users distracts more than circular and elliptical trajectory farther from the user’s face	No significant difference
	Adequacy	Elliptical trajectory far from the user and circular trajectory are more adequate than elliptical trajectory closer to the user’s face	Elliptical trajectory far from the user more adequate than circular and elliptical trajectory closer to the user’s face

## Data Availability

The data presented in this study are available on request from the corresponding author.

## Abbreviations

The following abbreviations are used in this manuscript:

GPI	Generalised Proportional Integral
HRI	Human-Robot Interaction
IQR	Interquartile Range
MQTT	Message Queue Telemetry Transport
SD	Standard Deviation
UAV	Unmanned Aerial Vehicle
VR	Virtual Reality
